# Complete genome sequencing of *Pandoraea pnomenusa* RB38 and Molecular Characterization of Its *N*-acyl homoserine lactone synthase gene *ppnI*

**DOI:** 10.7717/peerj.1225

**Published:** 2015-08-27

**Authors:** Yan-Lue Lim, Robson Ee, Kah-Yan How, Siew-Kim Lee, Delicia Yong, Kok Keng Tee, Wai-Fong Yin, Kok-Gan Chan

**Affiliations:** 1 Division of Genetics and Molecular Biology, Faculty of Science, Institute of Biological Sciences, University of Malaya, Kuala Lumpur, Malaysia; 2 Department of Medicine, Faculty of Medicine, University of Malaya, Kuala Lumpur, Malaysia

**Keywords:** *N*-acyl homoserine lactone (AHL), Miseq, Quorum sensing, PacBio, Whole genome mapping (WGM), *ppnR*, Cell-to-cell communication, Opgen, *ppnI*

## Abstract

In this study, we sequenced the genome of *Pandoraea pnomenusa* RB38 using Pacific Biosciences RSII (PacBio) Single Molecule Real Time (SMRT) sequencing technology. A pair of cognate *luxI/R* homologs was identified where the *luxI* homolog, *ppnI,* was found adjacent to a *luxR* homolog, *ppnR1*. An additional orphan *luxR* homolog, *ppnR*2, was also discovered. Multiple sequence alignment and phylogenetic analysis revealed that *ppnI* is an *N*-acyl homoserine lactone (AHL) synthase gene that is distinct from those of the nearest phylogenetic neighbor *viz. Burkholderia* spp. High resolution tandem mass spectrometry (LC-MS/MS) analysis showed that *Escherichia coli* BL21 harboring *ppnI* produced a similar AHL profile (*N*-octanoylhomoserine lactone, C8-HSL) as *P. pnomenusa* RB38, the wild-type donor strain, confirming that PpnI directed the synthesis of AHL in *P. pnomenusa* RB38. To our knowledge, this is the first documentation of the *luxI/R* homologs of the genus *Pandoraea*.

## Introduction

The theory of “quorum sensing” (QS) was coined in the late nineties describing bacterial cell-to-cell communication for the regulation of various genes (Bainton et al., 1992; Miller & Bassler, 2001; Schauder & Bassler, 2001). This communication is accomplished through the secretion and detection of small hormone-like chemical molecules known as autoinducers which facilitate intra- and inter-species microbial communication. There are different classes of autoinducers where upon reaching a threshold concentration, these signaling molecules activate and stimulate a wide variety of gene expression (Davies et al., 1998; Williams et al., 2007). The most studied QS molecule is *N*-acyl homoserine lactone (AHL) which is secreted by Gram-negative proteobacteria especially in the alpha-, beta- and gamma-proteobacteria subdivisions. AHL typically consists of a homoserine lactone moiety (Williams et al., 2007) and an *N*-acyl side chain with various chain length (C4–C18), a degree of saturation at C3 position and a presence of a hydroxy-, oxo- or no substituent at the C3 position (Chhabra et al., 2005). AHL synthase and receptor protein known as LuxI homolog and LuxR homolog respectively, are two typical principal protein families in AHLs QS system. Briefly, in this QS system, AHLs are secreted by LuxI homologs until a threshold concentration of AHL is attained before they bind to LuxR homologs and subsequently activate a cascade of QS-regulated gene expression (Fuqua, Parsek & Greenberg, 2001; Swift et al., 2001; Swift et al., 1996).

The name *Pandorea* originated from the term “Pandora’s box” which refers to the source of all evil in Greek mythology. Predominantly isolated from cystic fibrosis (CF) patients, *Pandoraea* species were also recovered from other clinical specimens and soil environment samples (Coenye et al., 2000; Daneshvar et al., 2001). Clinical manifestations of this terrorizing pathogen revolved around nosocomial infections with its capability to deteriorate lung function (Caraher et al., 2008; Costello et al., 2011; Stryjewski et al., 2003) and even cause multiple organ impairment (Stryjewski et al., 2003). However, the detailed mechanism of its colonization remains unknown despite emerging clinical documentations of this respiratory pathogen (Atkinson et al., 2006; Daneshvar et al., 2001; Stryjewski et al., 2003). To date, *Pandoraea* sp. is recognized as one of the lesser studied CF pathogens that requires further investigations particularly in its bacterial pathogenicity (Callaghan & McClean, 2012). To aggravate the situation, *Pandoraea* spp. are often misidentified in many clinical laboratories, leading to the lack of clinical documentation on its virulence potential (Hogardt et al., 2009). On the other hand, *Pandoraea* spp. have considerable attractions in biotechnological applications with various degradation abilities such as lignin degradation (Shi et al., 2013), polychlorinated biphenyls (PCBs) biodegradation (Dhindwal et al., 2011) and sulphur oxidation (Anandham et al., 2008).

Understanding of *Pandoraea* spp. at the genomic level is relatively superficial where majority of the literatures focuses firstly on usage of genotypic data to facilitate in accurate genus- and species-level identification (Coenye et al., 2001; Coenye & LiPuma, 2002) and secondly on their biotechnological potential (Schneider, Queenan & Bauernfeind, 2006; Jiang et al., 2009; Colbert et al., 2013; Ee et al., 2015). Furthermore, to date, inclusive of our recent report on the QS activity in *P. pnomenusa* RB38 (Ee et al., 2014b), there are only three publications about the documentation of the QS activity in *Pandoraea* spp. (Han-Jen, Wai-Fong & Kok-Gan, 2013; Chan et al., 2015). However, no detailed description or characterization of the QS genes in this genus have been performed. Hence, we sought to identify the presence of the AHL synthase in the genome of *P. pnomenusa* RB38 by sequencing its complete genome and further analysing the genes. As QS is well-known to regulate the expression of various genes such as virulence factors, identification of the LuxI/R homologs will be useful for further investigations of the QS-regulated gene expression. To our best knowledge, this is the first documentation of the QS system in the genus of *Pandoraea*.

## Methods

### Bacterial strains and culture conditions

Lysogeny medium (LBm) (Scharlau, Spain) was used as the only culture medium in the experiment. The AHL biosensors used in this experiment were *Chromobacterium violaceum* CV026, *Escherichia coli* [pSB401] and *E. coli* [pSB1142] while *Erwinia carotovora* GS101 and *E. carotovora* PNP22 were used as the positive and negative control for screening of AHL production. All isolates were cultured routinely in LBm broth or LBm agar plates at 28 °C with exception of *E. coli* [pSB401], *E. coli* [pSB1142] and *E. coli* BL21(DE3)pLysS, which were cultured aerobically at 37 °C.

### Complete genome sequencing, assembly and annotation

Complete genome sequencing was performed using Pacific Biosciences (PacBio) RS II Single Molecule Real Time (SMRT) sequencing technology (Pacific Biosciences, Menlo Park, CA) as described previously (Chan, Yin & Lim, 2014; Ee et al., 2014c). Briefly, the prepared 10-kb template library was sequenced on 4 single molecule real time (SMRT) cells using P4-C2 chemistry. *De novo* assembly was performed by filtering insert reads using RS_filter protocol (version 2.1.1) prior to assembly with Hierarchical Genome Assembly Process (HGAP) workflow in SMRT portal (version 2.1.1). Gene prediction was conducted using Prodigal version 2.60 (Hyatt et al., 2010).

Functional annotation of the predicted open reading frames (ORFs) was performed using the Rapid Annotation using Subsystem Technology (RAST) server (http://rast.nmpdr.org/rast.cgi). Classic RAST was selected as the annotation scheme whereas RAST gene caller (FIGfam release 70) was used as the gene caller. In addition, the genome was also annotated using Prokka (Seemann, 2014) and NCBI Prokaryotic Genome Annotation Pipeline (PGAP) (Version 2) (http://www.ncbi.nlm.nih.gov/genome/annotation_prok/), where default settings were used. The annotation predictions from the three pipelines were used in combination following the majority voting method to perform *in silico* identification of QS genes. The annotation predictions were manually evaluated and only genes predicted with consensus from two or more annotation pipelines were trusted in order to provide gene identification with high confidence.

For sequence-based genotypic identification, average nucleotide identity (ANI) values were calculated using the ANI calculator from Kostas Lab (http://enve-omics.ce.gatech.edu/ani/) whereas the 16S rRNA gene sequence which was retrieved using RNAmmer server (http://www.cbs.dtu.dk/services/RNAmmer/) was queried against the EzTaxon database (http://www.ezbiocloud.net/eztaxon).

### Whole genome optical mapping

Whole genome optical mapping was performed using OpGen Argus^®^ system (OpGen, Gaithersburg, MD) according to the manufacturer’s instructions. High molecular weight DNA was isolated from a single colony of sample strain using Argus High Molecular Weight (HMW) DNA Isolation Kit. DNA quality and concentration were determined using the Argus QCard kit. Single DNA molecules were then flowed through a microfluidic channel that was formed by Channel Forming Device (CFD) and were immobilized on a charged glass surface. By using the Enzyme Chooser software, BamHI was selected as the optimal restriction endonuclease for *P. pnomenusa* RB38 based on the FASTA-formatted sequence generated from PacBio RS II sequencing technology. The DNA molecules were digested on the glass surface to maintain the fragment order and were then stained with fluorescence dye. The image of the DNA fragments was captured using fluorescence microscopy and fully automated image-acquisition software. The single-molecule maps were assembled by overlapping DNA fragment patterns to produce a whole genome map with a minimum of 30× coverage. The whole genome map was aligned with PacBio FASTA-formatted sequences using the sequence placement tool in the MapSolver software (OpGen, Gaithersburg, MD).

### Identification of putative *luxI/R*-type QS genes

The predicted open reading frames (ORFs) were further annotated by comparing against NCBI-NR (ftp://ftp.ncbi.nlm.nih.gov/blast/db/) and Uniprot databases (http://www.uniprot.org/) to locate the AHL synthase (*ppnI*) and the AHL receptor protein (*ppnR*). The predicted proteome of *ppnI*/*R* were also further queried against NCBI conserved domain database (Marchler-Bauer et al., 2015) to confirm the authenticity of these putative QS genes.

Furthermore, phylogenetic trees of putative PpnI and PpnR were constructed using MEGA5 (Tamura et al., 2011). The putative translated product of *ppnI*/*R* was searched against NCBI non-redundant protein sequence (nr) database *via* the BLAST program (http://blast.ncbi.nlm.nih.gov/Blast.cgi) and homologous sequences were selected as reference sequences. The ClustalW method was selected to perform multiple alignment of the sequences whereas the neighbor-joining method (Saitou & Nei, 1987) with bootstrap test (1,000 replicates) was used to compute the phylogenetic tree. The following reference sequences were used to construct the phylogenetic tree (accession numbers in parentheses): LuxI *Burkholderia thailandensis* (WP006027437.1), LuxI *Burkholderia pseudomallei* (WP004532910.1), LuxI *Burkholderia oklahomensis* (WP010118441.1), LuxI *Burkholderia multivorans* (WP006396755.1), LuxI *Burkholderia cenocepacia* (WP015877501.1), LuxI *Burkholderia glumae* (WP015877501.1), LuxI *Ralstonia solanacearum* (WP020747102.1), LuxI *Aeromonas veronii* (AKK25355.1), PpnI *Pandoraea pnomenusa* RB38 (AHN77101.1), LuxR *Burkholderia stabilis* (AAG61132.1), LuxR *Burkholderia cepacia* (KER73646.1), LuxR *Burkholderia ambifaria* (WP006762266.1), LuxR *Burkholderia multivorans* (WP006403200.1), LuxR *Burkholderia dolosa* (WP006766136.1), LuxR *Burkholderia glumae* (WP017424156.1), LuxR *Ralstonia solanacearum* (WP013204747.1), LuxR *Aeromonas caviae* (KEP91903.1), PpnR1 *Pandoraea pnomenusa* RB38 (AHN77102.1) and PpnR2 *Pandoraea pnomenusa* RB38 (WP023594793.1).

Subsequently, the putative *ppnI* sequence was cloned into pUC57 vector (GeneScript, Piscataway, NJ) prior to cloning into pGS-21a expression vector. The resulting pGS-21a::*ppnI* plasmid was transformed into competent *E. coli* BL21(DE3)pLysS. Ampicillin (100 µg/ml) and chloramphenicol (34 µg/ml) (CalBioChem, Merck Millipore, Billerica, MA) were added to the growth medium to select the transformant.

### Screening of AHL production

Preliminary screening of AHL was performed by streaking transformed *E. coli* with the gene of interest against *C. violaceum* CV026 biosensor prior to 37 °C overnight incubation. *E. coli* harboring only  vector pGS-21a without the gene of interest was included as negative control.

AHL extraction was performed as previously described (Ee et al., 2014a). Briefly, spent supernatant of recombinant *E. coli* with the gene of interest was extracted twice with an equal volume of acidified ethyl acetate (0.1% v/v glacial acetic acid) and the organic layer was completely desiccated (Ortori et al., 2011). AHL profile was confirmed using LC-MS/MS triple quadrupole mass spectrometry (Agilent 1290 Infinity LC and Agilent 6490 Triple Quadrupole LC/MS systems, Agilent Technologies, Santa Clara, California, USA) as described previously (Ee et al., 2014a; Lim et al., 2014). AHL detection was performed using precursor ion mode where the precursor ion *m*/*z* value was scanned from 80 to 400. Agilent MassHunter software was used for data analysis.

### Thin layer chromatography

Thin layer chromatography was conducted with loading of 25 µL of extracted AHLs (in 100 µL of ACN) on activated reverse phase C18 TLC plates (TLC aluminium sheets 20 cm × 20 cm, RP-18 F254s, Merck, Darmstadt, Germany) (Shaw et al., 1997). Synthetic AHLs of *N*-octanoyl-_*L*_-homoserine lactone (C8-HSL) (Sigma–Aldrich, St Louis, Missouri, USA) were included as positive control and the chromatography was performed in (v/v) 60% methanol: 40% water volume. Once completed, the TLC plate was air-dried and overlaid with soft agar seeded with overnight culture of CV026 biosensor and incubated overnight (Chen et al., 2013; Lim et al., 2014).

## Results and Discussion

### Complete genome sequencing

In this study, PacBio RSII SMRT sequencing technology was used as the sequencing platform in which the genome of *P. pnomenusa* RB38 was assembled into a single contig (GenBank accession number CP007506.1). With an average coverage of 190-fold, 4755 ORFs were revealed in the 5.3797 Mb complete genome of *P. pnomenusa* RB38. By using Gepard (Krumsiek, Arnold & Rattei, 2007), a dot matrix analysis was performed on the FASTA formatted sequence file of the genome which confirmed the circular topology of the assembly (Fig. S1).

The complete genome was then validated using OpGen whole genome map processed with restriction enzyme, BamHI (Fig. S2). Genome Optical Mapping is commonly used as one of the laboratory techniques to provide a structural scaffold for contigs orientation as well as to visually identify errors in genome assemblies by using constructed whole genome optical restriction maps (Nagarajan, Read & Pop, 2008). Perfect alignment of the whole genome map (5.146 Mb) constructed with the complete genome assembly of *P. pnomenusa* RB38 confirmed the accuracy of the finished genome sequence.

### Sequence-based genotypic identification analysis

*Pandoraea* spp. belong to the beta-subclass of Proteobacteria with *Burkholderia* and *Ralstonia* as the closest neighbors (Coenye et al., 2000). In clinical microbiology laboratories, *Pandoraea* spp. are often misidentified as *Burkholderia cepacia* complex (Bcc) or *Ralstonia* spp. or initially reported as non-fermentative Gram-negative bacilli (Aravena-Román, 2008; Coenye et al., 2001). Initial annotation of *P*. *pnomenusa* RB38 complete genome using Rapid Annotation using Subsystem Technology (Version 4.0) (http://rast.nmpdr.org/rast.cgi) misidentified *Burkholderia* sp. CCGE1001 as the closest relative. This can be explained by the limited collection of 627 bacterial genome in RAST database at the time of writing. However, isolate identification performed in a previous study using 16S rDNA sequencing and Matrix-assisted Laser Desorption Ionization Time-of-Flight Mass Spectrometry (MALDI-TOF MS) identified strain RB38 as *P*. *pnomenusa* (Ee et al., 2014b).

With the availability of the whole genome sequence data, we performed two sequence-based genotypic microbial identification analysis, namely comparison of 16S rRNA gene sequence against type strain database using EzTaxon e-analysis and average nucleotide identity (ANI) analysis in order to evaluate the accuracy of these genotypic methods in identification of *Pandoraea* species. Firstly, EzTaxon e-analysis inferred from the 16S rRNA gene sequence of *P. pnomenusa* RB38 indicated that this strain clustered with its corresponding type strain, *P. pnomenusa* DSM-16536^*T*^ (AY268170) with pairwise similarity value of 99.86% (Table S1). Furthermore, genome comparison between *P. pnomenusa* RB38 and *P. pnomenusa* DSM-16536^*T*^ also generated an ANI value of 99.32% whereas genome comparison with other in-house sequenced *Pandoraea* type species provided ANI value of less than 86% (Table 1). The high pairwise similarity value (pairwise similarity cutoff value: 98.65%) (Kim et al., 2014) and ANI value (ANI cutoff value for species circumscription: 96%) (Richter & Rosselló-Móra, 2009) confirmed the species assignment of *P. pnomenusa* RB38 unequivocally.

**Table 1:** Average Nucleotide Identity (ANI) analysis. Genome comparison of *P. pnomenusa* RB38 and other *Pandoraea* type species.

	*1*	*2*	*3*	*4*	*5*	*6*	*7*	*8*	*9*
*1*	100	99.32	85.06	84.50	84.43	83.97	84.71	78.57	84.67
*2*	99.32	100	85.49	84.50	84.55	83.92	84.61	78.62	84.61
*3*	85.06	85.49	100	84.68	84.60	84.33	84.80	78.79	84.73
*4*	84.50	84.50	84.68	100	84.55	83.80	92.39	78.37	85.56
*5*	84.43	84.55	84.60	84.55	100	84.15	84.68	78.19	84.44
*6*	83.97	83.92	84.33	83.80	84.15	100	83.99	78.24	83.90
*7*	84.71	84.61	84.80	92.39	84.68	83.99	100	78.35	85.90
*8*	78.57	78.62	78.79	78.37	78.19	78.24	78.35	100	78.25
*9*	84.67	84.61	84.73	85.56	84.44	83.90	85.90	78.25	100

**Notes.**

Strains: 1, *Pandoraea pnomenusa* RB38; 2, *Pandoraea pnomenusa* DSM 16536^T^; 3, *Pandoraea pulmonicola* LMG 18106^T^; 4, *Pandoraea sputorum* LMG 18819^T^; 5, *Pandoraea apista* LMG 16407^T^; 6, *Pandoraea norimbergensis* DSM 11628^T^; 7, *Pandoraea oxalativorans* DSM 23570^T^; 8, *Pandoraea thiooxydans* LMG 24779^T^; 9, *Pandoraea vervacti* DSM 23571^T^.

The efficiency of genotypic identification methods in the identification of *P. pnomenusa* RB38 further supported the report from Coenye and colleagues (2001). They reported that genotypic identification should be used to complement phenotypic identification methods particularly in clinical microbiology laboratories to provide a high resolution identification for clinically important bacteria such as *Pandoraea* isolates. This will significantly reduce incidences of misidentification and hence improve epidemiological and clinical understanding of *Pandoraea* spp.

### Identification and *in silico* analysis of *luxI/R*-type QS genes

We previously reported the QS activity of *P. pnomenusa* RB38 (Ee et al., 2014b). In this study, we identified the putative *luxI* and *luxR*1 homologs from the annotated genome. Firstly, a 786 bp putative *N*-acyl homoserine lactone synthase (DA70_23485) (designated as *ppnI* gene) with the highest amino acid sequence similarity (100%) to a LuxI homolog of *Pandoraea* sp. RB-44 (AHB74553.1) was identified (Table S2). Conserved domain analysis of the predicted proteome of this gene indicated the presence of autoinducer synthase domain (PFAM signature: PF00765) which further confirmed that this gene is a genuine LuxI homolog.

**Figure 1:**
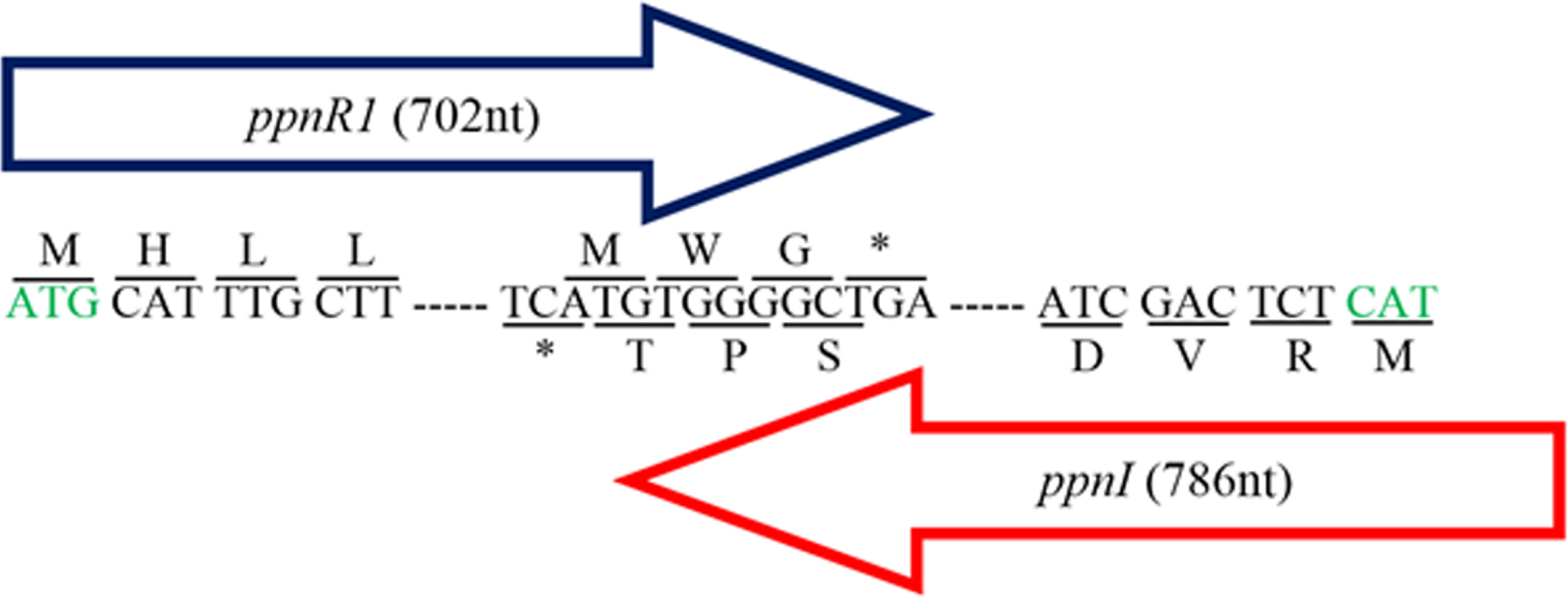
Gene map showing organization of *ppnR*1 (*luxR* homolog) and *ppnI* (*luxI* homolog). The direction of the arrows indicates the orientation of both genes where *ppnI* is in the 5′–3′ direction while *ppnR*1 is in the 3′–5′ direction. A line is used to indicate the nucleotide sequences and its respective amino acid sequence. Start codon, Methionine (M), is represented by a green font while the asterisk represents the stop codon (TGA). The *ppnR*1 and *ppnI* genes sequences have been deposited in GenBank database with GenBank accession numbers AHN77102.1 and AHN77101.1, respectively.

Additionally, a 702 bp putative cognate LuxR homolog (DA70_23490) (designated as *ppnR1* gene) located in close proximity and in a convergent transcriptional orientation to the *ppnI* gene was also manually identified (Fig. 1). Presence of LuxR homolog in close proximity to the LuxI homolog is commonly observed in the typical LuxI/LuxR-type QS circuit (Schaefer et al., 2013). The deduced amino acid sequence of *ppnR1* gene shows highest sequence similarity (100%) to LuxR homolog of *Pandoraea* sp. RB-44 (AHB74552.1) (Table S3). In order to confirm the authenticity of this putative LuxR homolog, the predicted protein sequence was scanned and confirmed to contain the universal conserved domain organization of LuxR proteins namely: the autoinducer binding domain (PFAM03472) and C-terminal DNA-binding domain of LuxR-like proteins (cd06170) (Choi & Greenberg, 1992; Fuqua, Parsek & Greenberg, 2001; Hanzelka & Greenberg, 1995).

Further search in the genome also indicated the presence of an additional putative *luxR* homologous gene (DA70_22525) (designated as *ppnR2*) which was not associated with a *luxI* homolog and is therefore referred to in this study as a putative orphan LuxR regulator. The PpnR2 protein shows 100% sequence similarity to LuxR homologs of multiple *Pandoraea* species (WP_023594793.1) (Table S4). Orphan LuxR is hypothesized to occur as a result of genes re-organizations, horizontal gene transfer or independent evolution of transcriptional regulatory circuits (Patankar & González, 2009b). Various studies have reported the identification of orphan LuxR in numerous bacteria and it was also found to interact with AHLs in regulating a variety of gene expression (Malott et al., 2009; Patankar & González, 2009a; Subramoni & Venturi, 2009).

**Figure 2:**
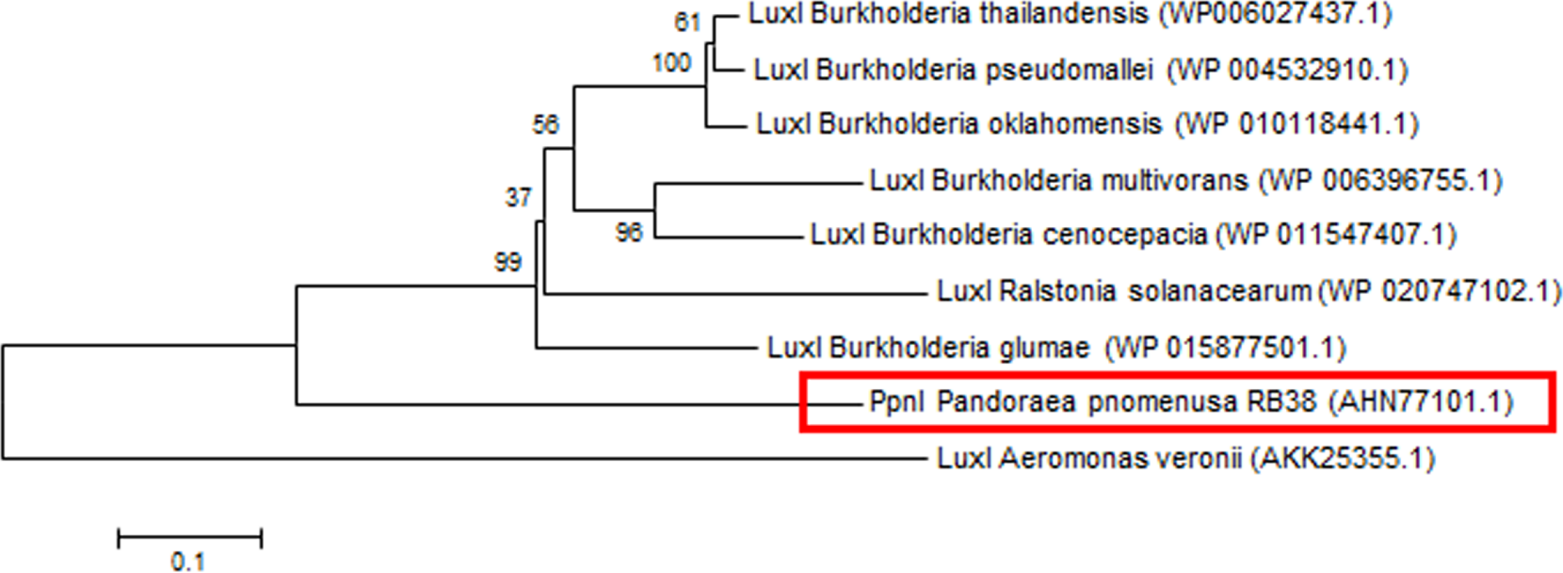
Phylogenetic tree of PpnI. Neighbor-Joining method (Saitou & Nei, 1987) was used in MEGA6 (Tamura et al., 2011) where bootstrap test (1,000 replicates) is shown next to the branches (Felsenstein, 1985).

**Figure 3:**
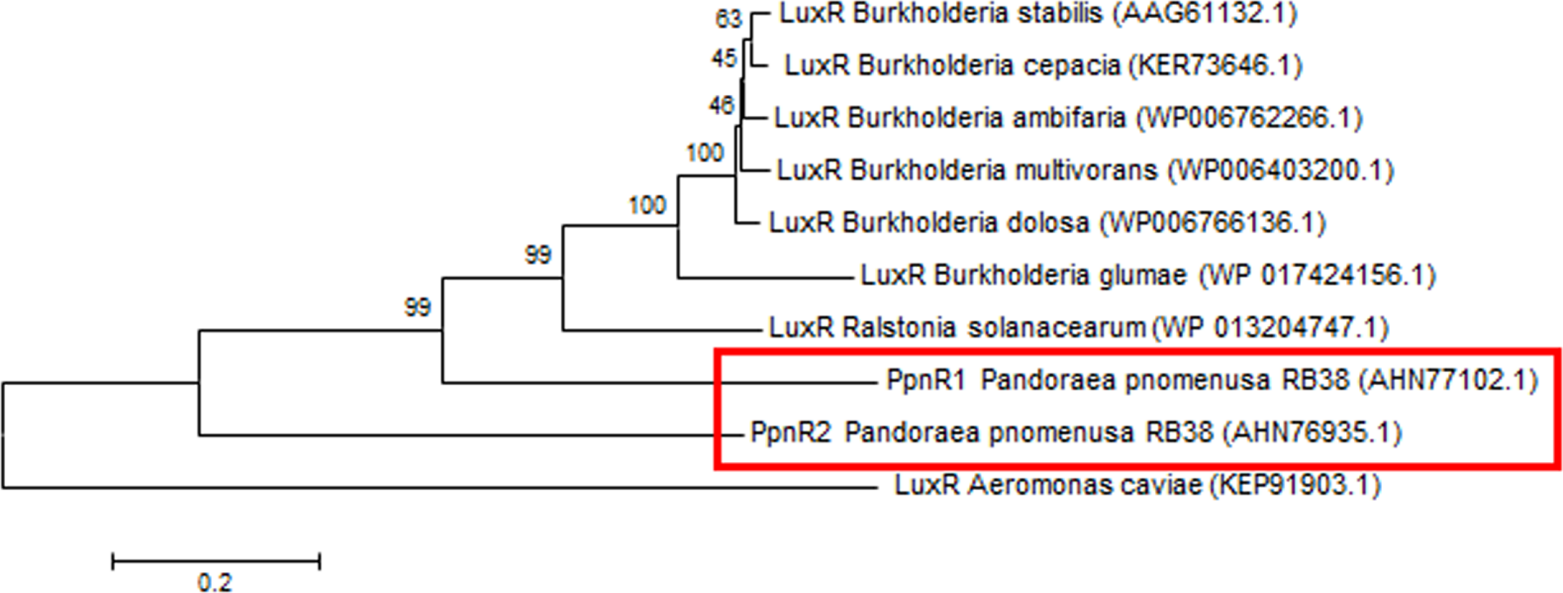
Phylogenetic tree of PpnR. Neighbor-Joining method (Saitou & Nei, 1987) was used in MEGA6 (Tamura et al., 2011) where bootstrap test (1,000 replicates) is shown next to the branches (Felsenstein, 1985).

Phylogenetic analyses performed indicated that both the PpnI/PpnR1 pair and the orphan PpnR2 are distant from LuxI or LuxR homologues of its closest phylogenetic neighbours, the *Burkholderia* and *Ralstonia* species (Figs. 2 and 3). Moreover , all three QS genes of *P. pnomenusa* RB38 exhibit low sequence similarity (less than 50%) to any previously characterised LuxI/LuxR homologues, namely BpsI (protein ID: AAQ90168.1) (Song et al., 2005); BpsI (protein ID: AAM21707.2) (Lumjiaktase et al., 2006); BmuR (Protein ID: AAK50054.2) (Yao, Zhou & Lessie, 2002); CepR (Protein ID: AAK70347.1) (Lutter et al., 2001); NmuR (Protein ID: AHB23331.1) (Gao et al., 2014); and VfqR (Protein ID: AGE97288.1) (Wang et al., 2013). This suggests that the QS genes of *P. pnomenusa* RB38 represent a new evolutionary branch of the QS system and can potentially have novel regulatory roles. To the best of our knowledge, this is the first documentation of LuxI/R homologs of the *P. pnomenusa* RB38.

### Functional study of putative *ppnI* gene

**Figure 4:**
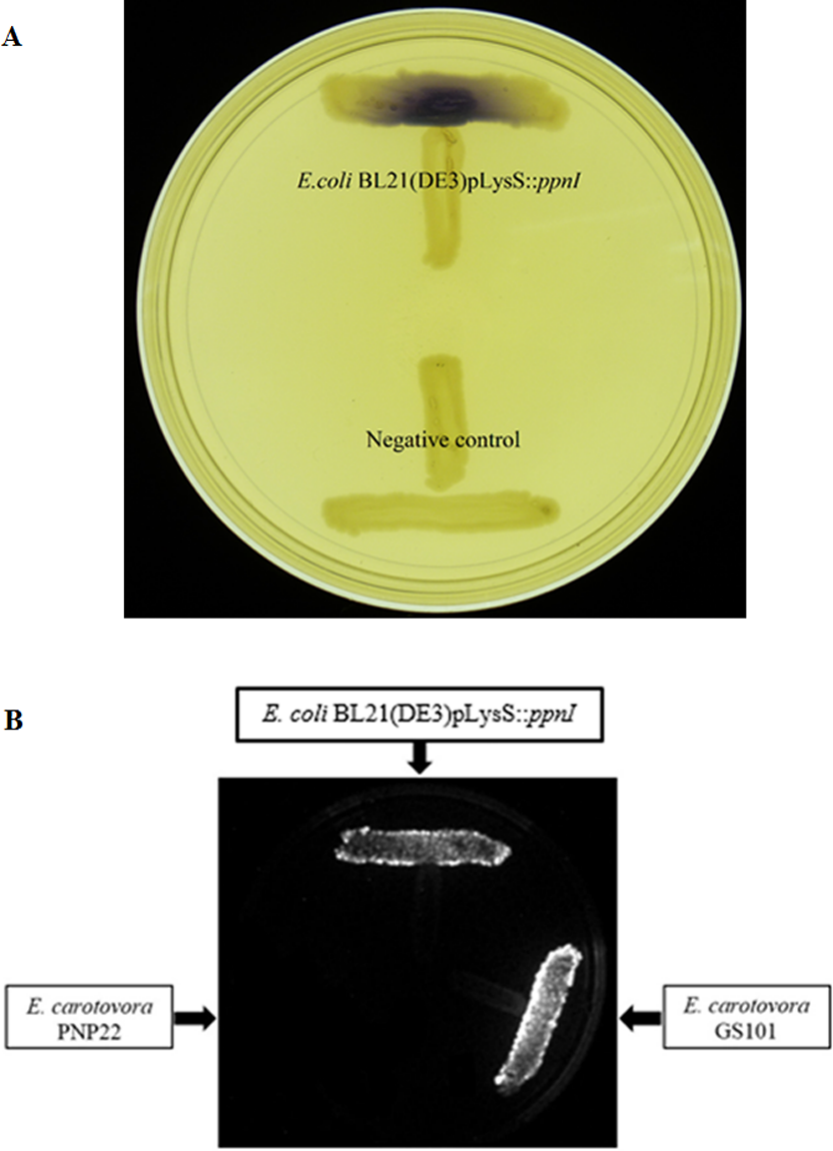
Cross Streaking bioassay. (A) CV026 bioassay. Purple pigmentation indicated secretion of short chain AHLs from *E. coli* BL21(DE3)pLysS::*ppnI*. Negative control namely *E. coli* harboring only vector pGS-21a without the gene of interest was included. (B) *E. coli* [pSB401] bioluminescence bioassay. Expression of bioluminescence activity in *E. coli* [pSB401] demonstrated the detection of short chain AHLs. *E. carotovora* GS101 and *E. carotovora* PNP22 served as the positive and negative controls, respectively.

**Figure 5:**
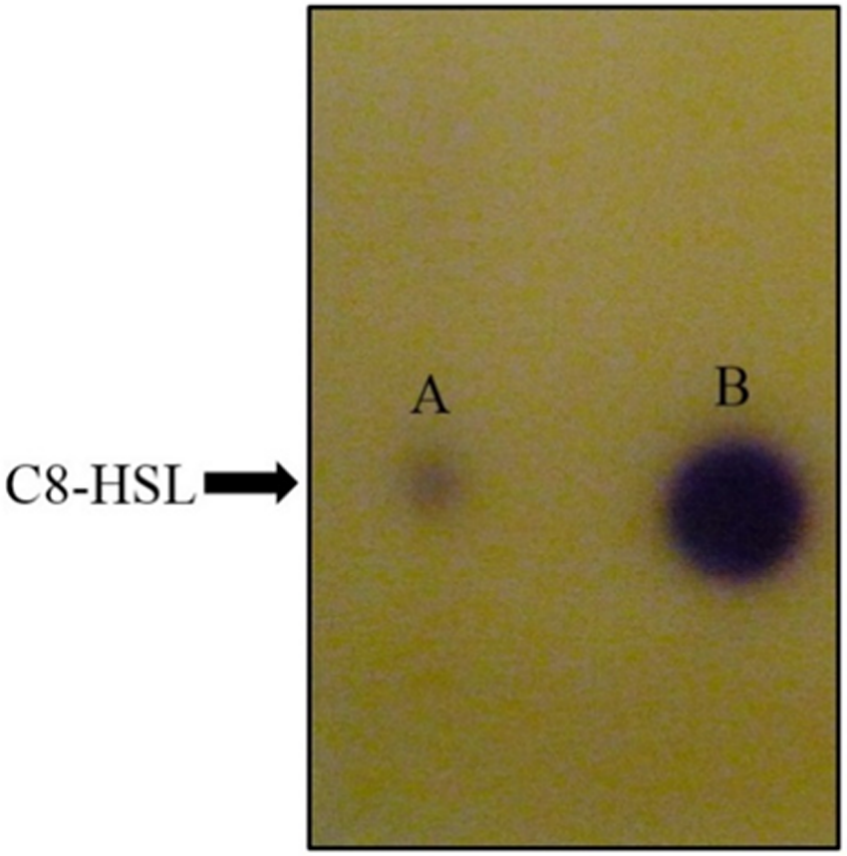
TLC bioassay overlaid with CV026 biosensor. Lane A: AHL extract of *E. coli* BL21(DE3)pLysS::*ppnI*; Lane B: Synthetic C8-HSL.

**Figure 6:**
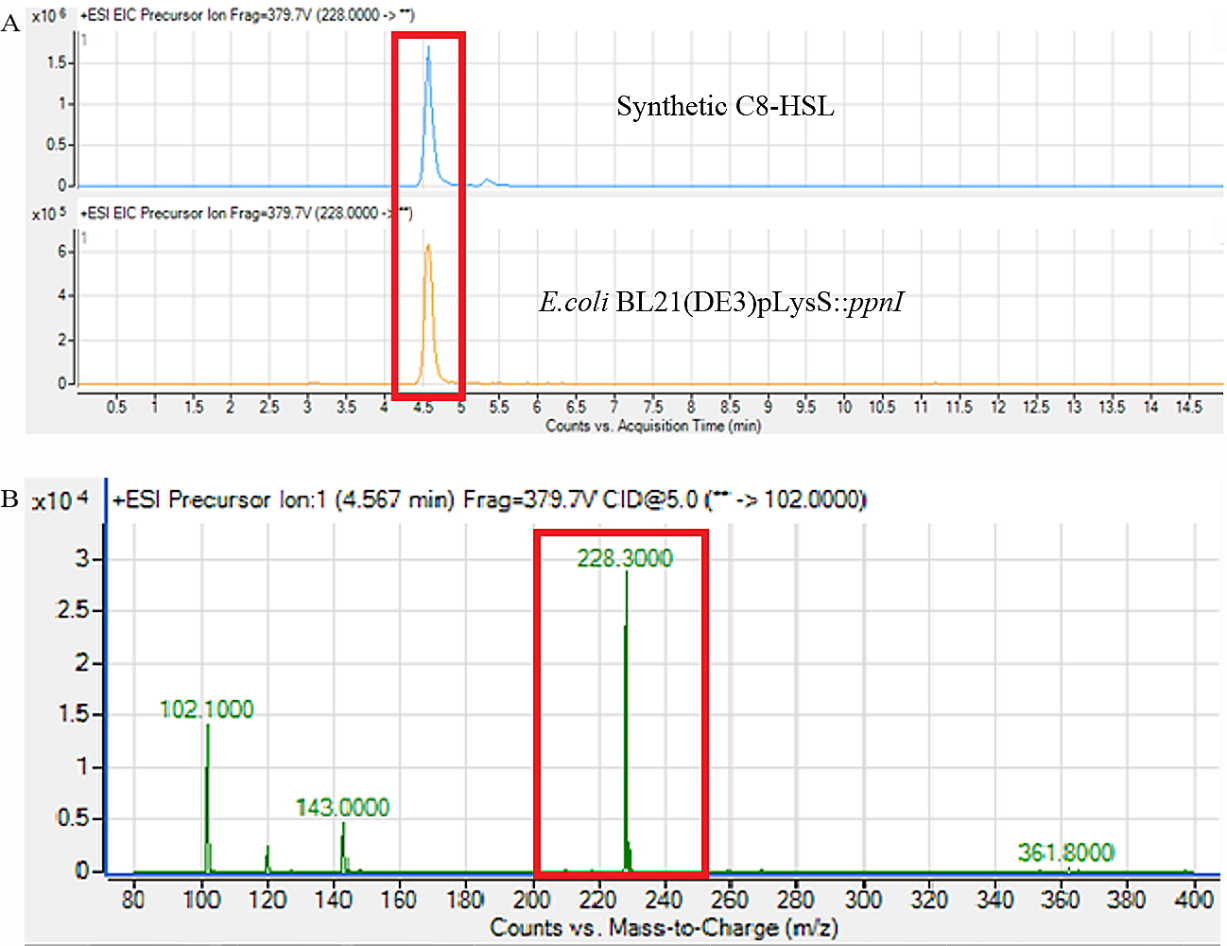
Mass spectrometry analysis of *E. coli* BL21(DE3)pLysS::*ppnI*. (A) showed that the retention time of C8-HSL produced by *E. coli* BL21(DE3)pLysS::*ppnI* was similar with the retention time of synthetic C8-HSL used as positive control. (B) showed the *m/z* value of 228.300 (C8-HSL) detected from the supernatant of *E. coli* BL21(DE3)pLysS::*ppnI* with the retention time of 4.567 min. The abundance percentage detected was 100%.

For functional studies, we cloned the putative *ppnI* into a pGS-21a expression vector and subsequently transformed the pGS-21a::*ppnI* plasmid into competent *E. coli* BL21(DE3)pLysS. AHL screening were performed using *C. violaceum* CV026 biosensor with *E. coli* BL21(DE3)pLysS::*ppnI*. The result of the cross-streak bioassay demonstrated activation of purple violacein secretion of *C. violaceum* CV026 (Fig. 4A) as well as bioluminescence activity of *E. coli* [pSB401] indicating the production of short chain AHLs by the *ppnI* gene (Fig. 4B). Besides that, formation of a sole purple violacein spot on CV026 lawn which corresponds to the same retention time of the synthetic C8-HSL suggested that the *ppnI* is responsible for the production of C8-HSL in *P. pnomenusa* RB38 (Fig. 5). The AHL profile of *ppnI* was further verified using LC-MS/MS mass spectrometry system and only C8-HSL was detected in the supernatant of recombinant *E. coli* BL21 suggesting that *ppnI* is indeed the functional LuxI synthase of *P. pnomenusa* RB38 (Fig. 6).

## Conclusion

We report the complete genome sequence of *P. pnomenusa* RB38 and the discovery of its AHL synthase, designated as *ppnI* gene and its LuxR homolog receptor, *ppnR* gene, as well as an additional orphan LuxR regulator, *ppnR*2 gene. Short chain AHL C8-HSL was detected in the spent culture supernatant of *E. coli* BL21(DE3)pLysS::*ppnI* which confirmed that *ppnI* gene is a functional AHL synthase. Furthermore, we have also confirmed the efficiency of genotypic identification methods in providing unambiguous species assignment for the *Pandoraea* species.

## Supplemental Information

10.7717/peerj.1225/supp-1 Figure S1 Dot plot graph constructed using Gepard (version 1.30) showing the comparison of *P. pnomenusa* RB38 linear assembly Presence of two same direction repeats region (not shown) at the ends of the assembly indicated the circular structure of this assembly.

10.7717/peerj.1225/supp-2 Figure S2 Sequence placement analysis performed using MapSolver™ alignment software Whole genome mapping data (top sequence) was compared against PacBio single contig genome (bottom sequence). Blue colour indicates similarity found in both two sequences. Whole genome mapping data confirmed that the whole genome sequencing of *P. pnomenusa* RB38 genome from PacBio sequencing platform is an accurately assembled complete genome.

10.7717/peerj.1225/supp-3 Table S1 Supplementary Table

10.7717/peerj.1225/supp-4 Table S2 Blast hits for PpnI (Top 15)

10.7717/peerj.1225/supp-5 Supplemental Information 5 Genbank Accession Numbers

10.7717/peerj.1225/supp-6 Table S3 Blast hits for PpnR1 (Top 15)

10.7717/peerj.1225/supp-7 Table S4 Blast hits for PpnR2 (top 15)

## Additional Information and Declarations

### DNA Deposition

The following information was supplied regarding the deposition of DNA sequences: All data have been deposited to GenBank. Genbank Accession number ppnI, ppnR1, and ppnR2 sequences are available in GenBank database with the accession numbers of AHN77101.1, AHN77102.1, and AHN76935.1, respectively.
