# Supplementary material for: Complete genome sequencing of Pandoraea pnomenusa RB38 and Molecular Characterization of Its N-acyl homoserine lactone synthase gene ppnI
Source: PeerJ. 2015 Aug 27;3:e1225. doi: 10.7717/peerj.1225 (PMC4556143; doi:10.7717/peerj.1225)
Supplement: Figure S1 — Presence of two same direction repeats region (not shown) at the ends of the assembly indicated the circular structure of this assembly. [file peerj-03-1225-s001.pdf]

Supplementary figure 1

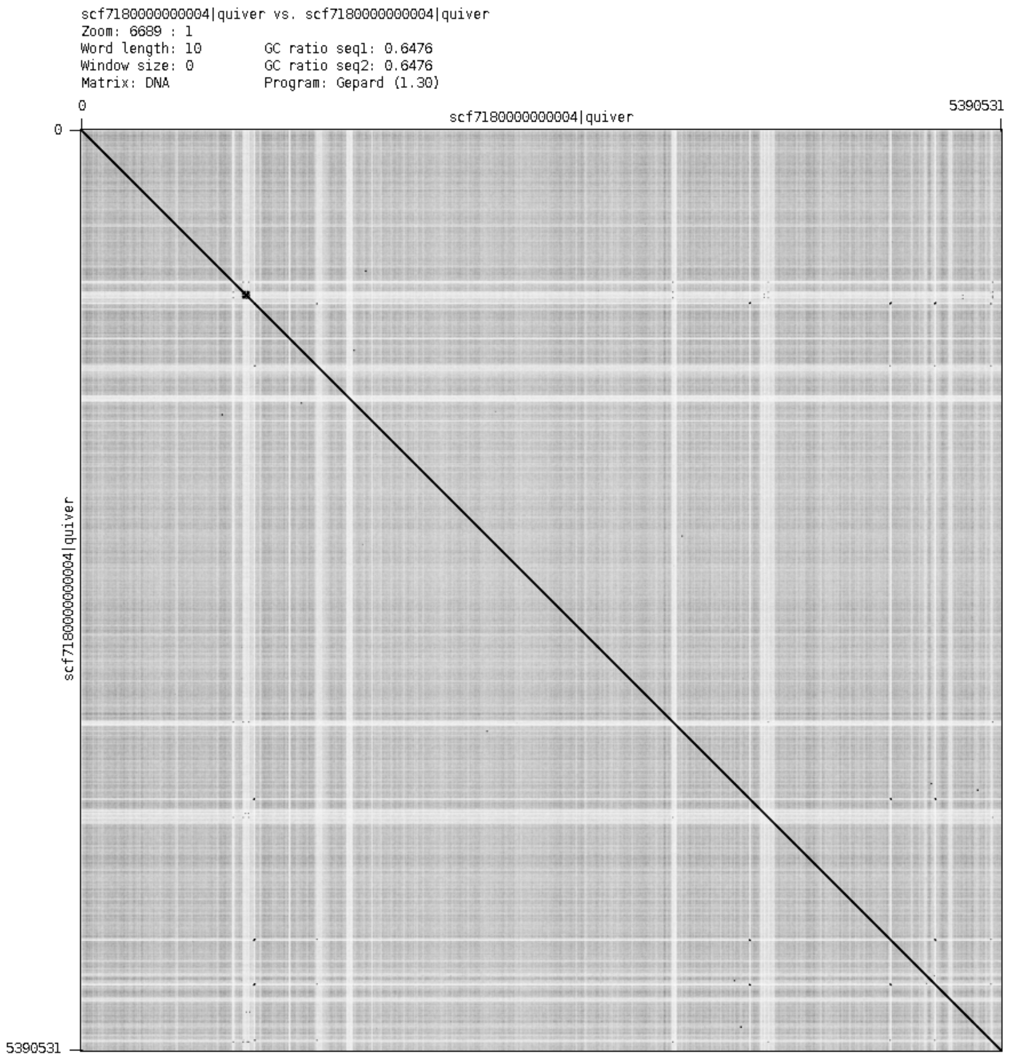

Supplementary figure 1. Dot plot graph constructed using Gepard (version 1.30) showing the comparison of *P. pnomenusa* RB38 linear assembly. Presence of two same direction repeats region (not shown) at the ends of the assembly indicated the circular structure of this assembly.
