# Supplementary material for: Complete genome sequencing of Pandoraea pnomenusa RB38 and Molecular Characterization of Its N-acyl homoserine lactone synthase gene ppnI
Source: PeerJ. 2015 Aug 27;3:e1225. doi: 10.7717/peerj.1225 (PMC4556143; doi:10.7717/peerj.1225)
Supplement: Figure S2 — Whole genome mapping data (top sequence) was compared against PacBio single contig genome (bottom sequence). Blue colour indicates similarity found in both two sequences. Whole genome mapping data confirmed that the whole genome sequencing of P. pnomenusa RB38 genome from PacBio sequencing platform is an accurately assembled complete genome. [file peerj-03-1225-s002.pdf]

Supplementary figure 2

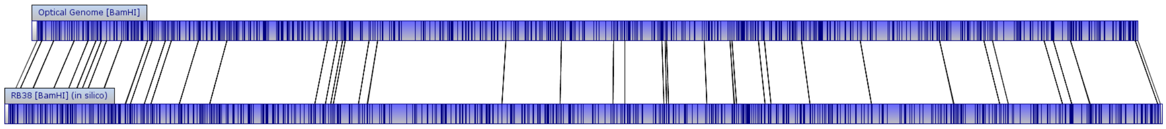

Supplementary figure 2. Sequence placement analysis performed using MapSolver™ alignment software. Whole genome mapping data (top sequence) was compared against PacBio single contig genome (bottom sequence). Blue colour indicates similarity found in both two sequences. Whole genome mapping data confirmed that the whole genome sequencing of *P. pnomenusa* RB38 genome from PacBio sequencing platform is an accurately assembled complete genome.
