# Supplementary material for: Complete genome sequencing of Pandoraea pnomenusa RB38 and Molecular Characterization of Its N-acyl homoserine lactone synthase gene ppnI
Source: PeerJ. 2015 Aug 27;3:e1225. doi: 10.7717/peerj.1225 (PMC4556143; doi:10.7717/peerj.1225)
Supplement: Table S1 [file peerj-03-1225-s003.pdf]

Rank	Name	Strain	Authors	Accession	Pairwise Similarity (%)	Diff/Total nt	Completeness (%)
1	*Pandoraea pnomenusa*	CCUG 38742(T)	Coenye et al. 2000	AY268170	99.86	2/1461	100
2	*Pandoraea faecigallinarum*	KOx(T)	Sahin et al. 2011	AB510956	99.78	3/1378	94.32
3	*Pandoraea pulmonicola*	CCUG 38759(T)	Coenye et al. 2000	AY268173	99.52	7/1461	100
4	*Pandoraea apista*	CCUG 38412(T)	Coenye et al. 2000	AY268172	99.45	8/1461	100
5	*Pandoraea vervacti*	NS15(T)	Sahin et al. 2011	AB510957	99.35	9/1376	94.31
6	*Pandoraea oxalativorans*	TA25(T)	Sahin et al. 2011	AB469785	99.2	11/1375	94.18
7	*Pandoraea sputorum*	LMG 18819(T)	Coenye et al. 2000	AF139176	99.18	12/1470	100
8	*Pandoraea norimbergensis*	CCUG 39188(T)	(Wittke et al. 1998) Coenye et al. 2000	AY268174	99.04	14/1461	100
9	*Pandoraea thiooxydans*	ATSB16(T)	Anandham et al. 2010	EF397578	97.23	39/1408	96.91
10	*Burkholderia pseudomultivorans*	LMG 26883(T)	Peeters et al. 2014	HE962386	96.05	58/1469	100
11	*Burkholderia stabilis*	LMG 14294(T)	Vandamme et al. 2000	AF148554	95.98	59/1469	100
12	*Burkholderia seminalis*	R-24196(T)	Vanlaere et al. 2008	AM747631	95.93	60/1475	100
13	*Burkholderia anthina*	R-4183(T)	Vandamme et al. 2002	AJ420880	95.92	60/1471	100
14	*Burkholderia caledonica*	NBRC 102488(T)	Coenye et al. 2001	BAYE01000 050	95.9	61/1489	100
15	*Burkholderia phytofirmans*	PsJN(T)	Sessitsch et al. 2005	CP001053	95.9	61/1489	100
16	*Burkholderia unamae*	MTl-641(T)	Caballero-Mellado et	AY221956	95.85	61/1471	100

			al. 2004				
17	*Burkholderia cepacia*	ATCC 25416(T)	(Palleroni and Holmes 1981) Yabuuchi et al. 1993	AXBO01000009	95.84	62/1491	100
18	*Burkholderia ambifaria*	AMMD(T)	Coenye et al. 2001	CP000442	95.84	62/1491	100
19	*Burkholderia oklahomensis*	C6786(T)	Glass et al. 2006	ABBG01000575	95.84	62/1491	100
20	*Burkholderia jiangsuensis*	MP-1(T)	Liu et al. (in press)	KJ400396	95.83	62/1488	100
21	*Burkholderia pyrrocinia*	LMG 14191(T)	(Imanaka et al. 1965) Vandamme et al. 1997	U96930	95.82	60/1434	97.62
22	*Burkholderia diffusa*	R-15930(T)	Vanlaere et al. 2008	AM747629	95.81	62/1478	100
23	*Burkholderia arboris*	R-24201(T)	Vanlaere et al. 2008	AM747630	95.8	62/1477	100
24	*Burkholderia cenocepacia*	J2315(T)	Vandamme et al. 2003	AM747720	95.77	63/1491	100
25	*Burkholderia metallica*	R-16017(T)	Vanlaere et al. 2008	AM747632	95.74	63/1478	100
26	*Burkholderia glumae*	LMG 2196(T)	(Kurita and Tabei 1967) Urakami et al. 1994	AMRF01000003	95.71	64/1491	100
27	*Burkholderia rinojensis*	A396(T)	Cordova-Kreylos et al. 2013	KF650996	95.7	64/1490	100
28	*Burkholderia grimmiae*	R27(T)	Tian et al. 2013	JFHE01000086	95.7	64/1489	100
29	*Burkholderia terrestris*	LMG 22937(T)	Vandamme et al. 2013	HE981726	95.67	64/1478	100
30	*Burkholderia tropica*	Ppe8(T)	Reis et al. 2004	AJ420332	95.66	61/1406	96.63
31	*Burkholderia lata*	383(T)	Vanlaere et al. 2009	CP000150	95.64	65/1491	100
32	*Burkholderia latens*	R-5630(T)	Vanlaere et al. 2008	AM747628	95.61	65/1480	100
33	*Burkholderia sacchari*	IPT101(T)	Brämer et al. 2001	AF263278	95.57	66/1489	100

34	*Burkholderia vietnamiensis*	LMG 10929(T)	Gillis et al. 1995	AF097534	95.5	67/1489	100
35	*Burkholderia rhizoxinica*	HKI 454(T)	Partida-Martinez et al. 2007	FR687359	95.5	67/1489	100
36	*Burkholderia sediminicola*	HU2-65W(T)	Lim et al. 2008	EU035613	95.45	67/1473	100
37	*Burkholderia bannensis*	NBRC 103871(T)	Aizawa et al. 2011	BAYA01000085	95.44	68/1491	100
38	*Burkholderia multivorans*	ATCC BAA-247(T)	Vandamme et al. 1997	ALIW01000278	95.44	68/1491	100
39	*Burkholderia oxyphila*	NBRC 105797(T)	Otsuka et al. 2011	BAYD01000210	95.44	68/1491	100
40	*Burkholderia cordobensis*	LMG 27620(T)	Draghi et al. 2014	HG324048	95.44	67/1468	100
41	*Burkholderia thailandensis*	E264(T)	Brett et al. 1998	CP000086	95.43	68/1489	100
42	*Burkholderia telluris*	LMG 22936(T)	Vandamme et al. 2013	HE981727	95.4	68/1478	100
43	*Burkholderia zhejiangensis*	OP-1(T)	Lu et al. 2012	JFHD01000081	95.3	70/1489	100
44	*Burkholderia nigropunctata*	19750521	van Oevelen et al. 2004	AY277698	95.3	70/1488	100
45	*Burkholderia dilworthii*	WSM3556(T)	De Meyer et al. 2014	HQ698908	95.27	70/1479	100
46	*Burkholderia caryophylli*	ATCC 25418(T)	(Burkholder 1942) Yabuuchi et al. 1993	AB021423	95.23	69/1448	98.57
47	*Burkholderia denitrificans*	KIS30-44(T)	Lee et al. 2013	GU171384	95.2	68/1416	97.53
48	*Burkholderia xenovorans*	LB400(T)	Goris et al. 2004	CP000270	95.16	72/1489	100
49	*Burkholderia sordidicola*	S5-B(T)	Lim et al. 2003	AF512826	95.16	71/1468	99.93

50	*Burkholderia dolosa*	LMG 18943(T)	Vermis et al. 2004	JX986970	95.08	73/1483	100
51	*Burkholderia phymatum*	STM815(T)	Vandamme et al. 2003	CP001043	94.9	76/1489	100
52	*Burkholderia nodosa*	R-25485(T)	Chen et al. 2007	AM284971	94.89	75/1467	100
53	*Burkholderia terrae*	KMY02(T)	Yang et al. 2006	AB201285	94.69	78/1469	100
54	*Burkholderia caballeronis*	TNe-841(T)	Martínez-Aguilar et al. 2014	EF139186	94.62	80/1487	100
55	*Burkholderia ferrariae*	NBRC 106233(T)	Valverde et al. 2006	BAYB01000079	94.16	87/1489	100
56	*Bordetella petrii*	DSM 12804(T)	von Wintzingerode et al. 2001	AM902716	91.54	126/1489	100
57	*Achromobacter animicus*	LMG 26690(T)	Vandamme et al. 2013	HE613448	91.25	129/1475	100
58	*Bordetella holmesii*	ATCC 51541(T)	Weyant et al. 1995	CP007494	91.18	134/1520	100
59	*Alcaligenes aquatilis*	LMG 22996(T)	Van Trappen et al. 2005	JX986974	90.75	137/1481	100
