# Supplementary material for: Complete genome sequencing of Pandoraea pnomenusa RB38 and Molecular Characterization of Its N-acyl homoserine lactone synthase gene ppnI
Source: PeerJ. 2015 Aug 27;3:e1225. doi: 10.7717/peerj.1225 (PMC4556143; doi:10.7717/peerj.1225)
Supplement: Table S2 [file peerj-03-1225-s004.pdf]

Supplementary table 2: Blast hits for PpnI (Top 15)

Predicted function	Microorganism	% identity (no. of similar aa[a]/total no.)	Accession Number
Acyl-homoserine lactone synthase	*Pandoraea* sp. RB-44	100%	AHB74553.1
Hypothetical protein	Multispecies: *Pandoraea*	100%	WP_029754786.1
Hypothetical protein	*Pandoraea pnomenusa*	99%	WP_041624254.1
Hypothetical protein	*Pandoraea vervacti*	71%	WP_044456583.1
Hypothetical protein	*Pandoraea faecigallinarum*	65%	WP_047905500.1
Hypothetical protein	*Pandoraea oxalativorans*	68%	WP_046292715.1
Hypothetical protein	*Pandoraea vervacti*	69%	AJP59868.1
Hypothetical protein	*Pandoraea sputorum*	59%	WP_039402528.
Acyl-homoserine lactone synthase	*Burkholderia oklahomensis*	43%	WP_010118441.1
Acyl-homoserine lactone synthase	*Burkholderia oklahomensis*	43%	WP_010108729.1
Acyl-homoserine lactone synthase	*Burkholderia pseudomallei*	43%	WP_038744068.1
Acyl-homoserine lactone synthase	*Burkholderia thailandensis*	43%	WP_006027437.1
Acyl-homoserine lactone synthase	*Burkholderia* sp. JPY347	44%	WP_026229463.1
Acyl-homoserine lactone synthase	*Burkholderia multivorans*	42%	WP_006396755.1
Acyl-homoserine lactone synthase	*Burkholderia pyrrocinia*	40%	WP_026043906.1

[a] aa, amino acid.
