# Supplementary material for: Complete genome sequencing of Pandoraea pnomenusa RB38 and Molecular Characterization of Its N-acyl homoserine lactone synthase gene ppnI
Source: PeerJ. 2015 Aug 27;3:e1225. doi: 10.7717/peerj.1225 (PMC4556143; doi:10.7717/peerj.1225)
Supplement: Supplemental Information 5 [file peerj-03-1225-s005.docx]

**Genbank Accession number**

*ppnI*, *ppnR*1, and *ppnR*2 sequences are available in GenBank database with the accession numbers of AHN77101.1, AHN77102.1, and AHN76935.1, respectively.
