# Supplementary material for: Complete genome sequencing of Pandoraea pnomenusa RB38 and Molecular Characterization of Its N-acyl homoserine lactone synthase gene ppnI
Source: PeerJ. 2015 Aug 27;3:e1225. doi: 10.7717/peerj.1225 (PMC4556143; doi:10.7717/peerj.1225)
Supplement: Table S3 [file peerj-03-1225-s006.docx]

**Supplementary table 3: Blast hits for PpnR1 (Top 15)**

| Predicted function | Microorganism | % identity  (no. of similar aa^a^/total no.) | Accession Number |
| --- | --- | --- | --- |
| *N*-acyl homoserine lactone transcriptional regulator | *Pandoraea pnomenusa* | 100% (233/233) | AHN77102.1 |
| LuxR family transcriptional regulator | *Pandoraea* sp. RB-44 | 99% (230/233) | AHB74552.1 |
| *N*-acyl homoserine lactone transcriptional regulator | *Pandoraea* sp. RB-44 | 99% (230/233) | AHI58916.1 |
| Hypothetical protein | *Pandoraea pnomenusa* | 100% (214/214) | WP_029754787.1 |
| Hypothetical protein | *Pandoraea pnomenusa* | 99% (212/214) | [WP_041624256.1](http://www.ncbi.nlm.nih.gov/protein/753868644?report=genbank&log$=prottop&blast_rank=5&RID=T9WJZ3YU015) |
| Hypothetical protein | *Pandoraea* sp. RB-44 | 99% (211/214) | [WP_036641605.1](http://www.ncbi.nlm.nih.gov/protein/738747831?report=genbank&log$=prottop&blast_rank=6&RID=T9WJZ3YU015) |
| Hypothetical protein | *Pandoraea* sp. E26 | 98% (210/214) | [WP_036656019.1](http://www.ncbi.nlm.nih.gov/protein/738762750?report=genbank&log$=prottop&blast_rank=7&RID=T9WJZ3YU015) |
| Hypothetical protein | [*Pandoraea faecigallinarum*](http://blast.ncbi.nlm.nih.gov/Blast.cgi#alnHdr_837947993) | 76% (177/232) | [WP_047908289.1](http://www.ncbi.nlm.nih.gov/protein/837947993?report=genbank&log$=prottop&blast_rank=8&RID=T9WJZ3YU015) |
| Hypothetical protein | [*Pandoraea oxalativorans*](http://blast.ncbi.nlm.nih.gov/Blast.cgi#alnHdr_809003655) | 76% (176/232) | [WP_046293945.1](http://www.ncbi.nlm.nih.gov/protein/809003655?report=genbank&log$=prottop&blast_rank=9&RID=T9WJZ3YU015) |
| Hypothetical protein | [*Pandoraea vervacti*](http://blast.ncbi.nlm.nih.gov/Blast.cgi#alnHdr_764685693) | 76% (176/232) | [WP_044458339.1](http://www.ncbi.nlm.nih.gov/protein/764685693?report=genbank&log$=prottop&blast_rank=10&RID=T9WJZ3YU015) |
| Hypothetical protein | [*Pandoraea sputorum*](http://blast.ncbi.nlm.nih.gov/Blast.cgi#alnHdr_746357867) | 69% (161/232) | [WP_039402529.1](http://www.ncbi.nlm.nih.gov/protein/746357867?report=genbank&log$=prottop&blast_rank=11&RID=T9WJZ3YU015) |
| LuxR family transcriptional regulator | [*Burkholderia pseudomallei*](http://blast.ncbi.nlm.nih.gov/Blast.cgi#alnHdr_740959450) | 48% (105/218) | [WP_038744072.1](http://www.ncbi.nlm.nih.gov/protein/740959450?report=genbank&log$=prottop&blast_rank=12&RID=T9WJZ3YU015) |
| LuxR family transcriptional regulator | [*Burkholderia pyrrocinia*](http://blast.ncbi.nlm.nih.gov/Blast.cgi#alnHdr_736006489) | 48% (105/218) | [WP_034180404.1](http://www.ncbi.nlm.nih.gov/protein/736006489?report=genbank&log$=prottop&blast_rank=13&RID=T9WJZ3YU015) |
| LuxR family transcriptional regulator | [*Burkholderia multivorans*](http://blast.ncbi.nlm.nih.gov/Blast.cgi#alnHdr_493447871) | 47% (106/225) | [WP_006403200.1](http://www.ncbi.nlm.nih.gov/protein/493447871?report=genbank&log$=prottop&blast_rank=14&RID=T9WJZ3YU015) |
| LuxR family transcriptional regulator | [*Mumia flava*](http://blast.ncbi.nlm.nih.gov/Blast.cgi#alnHdr_746296904) | 48% 9104/218) | [WP_039344021.1](http://www.ncbi.nlm.nih.gov/protein/746296904?report=genbank&log$=prottop&blast_rank=15&RID=T9WJZ3YU015) |

^a^ aa, amino acid.
