# Supplementary material for: Complete genome sequencing of Pandoraea pnomenusa RB38 and Molecular Characterization of Its N-acyl homoserine lactone synthase gene ppnI
Source: PeerJ. 2015 Aug 27;3:e1225. doi: 10.7717/peerj.1225 (PMC4556143; doi:10.7717/peerj.1225)
Supplement: Table S4 [file peerj-03-1225-s007.pdf]

Supplementary table 4: Blast hits for PpnR2 (top 15)

Predicted function	Microorganism	% identity (no. of similar aa[a]/total no.)	Accession Number
LuxR family transcriptional regulator	Multispecies: *Pandoraea*	100% (255/255)	WP_023594793.1
Hypothetical protein	*Pandoraea apista*	94% (240/255)	WP_042112508.1
Hypothetical protein	*Pandoraea pnomenusa*	93% (238/255)	WP_039375491.1
Hypothetical protein	*Pandoraea vervacti*	93% (236/255)	WP_044456713.1
Hypothetical protein	*Pandoraea faecigallinarum*	92% (234/255)	WP_047905625.1
Hypothetical protein	*Pandoraea sputorum*	92% (235/255)	WP_039400594.1
Hypothetical protein	*Pandoraea* sp. SD6-2	93% (236/255)	WP_010806955.1
Hypothetical protein	*Pandoraea oxalativorans*	92% (234/255)	WP_046292848.1
Hypothetical protein	*Pandoraea pulmonicola*	89% (228/255)	WP_039404904.1
Hypothetical protein	*Pandoraea* sp. B-6	88% (213/242)	WP_017233913.1
LuxR family transcriptional regulator	*Burkholderia pseudomallei*	43% (109/252)	WP_038788744.1
LuxR family transcriptional regulator	*Multispecies: Ralstonia*	42% (105/251)	WP_012434925.1
DNA-binding protein	*Ralstonia pickettii*	42% (105/251)	WP_004635427.1
Hypothetical protein	*Burkholderia cepacia*	43% (111/256)	WP_043189145.1
LuxR family transcriptional regulator	*Ralstonia pickettii*	41% (104/251)	WP_024973503.1

[a] aa, amino acid.
